# An audit of a combined rheumatology and orthopaedic foot and ankle surgery clinic

**DOI:** 10.1186/1757-1146-3-S1-O25

**Published:** 2010-12-20

**Authors:** Heidi Siddle, Michael Backhouse, Ray Monkhouse, Nick Harris, Philip Helliwell

**Affiliations:** 1 University of Leeds, Leeds, UK; 2 Leeds Teaching Hospitals NHS Trust, Leeds, UK

## Introduction

Failure of conservative care is often followed by orthopaedic intervention with foot surgery accounting for 1/3 of lower limb surgery in Rheumatoid Arthritis (RA). National guidelines encourage combined rheumatology & orthopaedic care.

## Methods

Rheumatology patients with persistent foot pain despite conservative care were referred to a combined rheumatology and orthopaedic clinic. Patients with RA completed the Leeds Foot Impact Scale (LFIS) and a 100mm pain VAS (pVAS) for each foot at baseline and follow up (FU).

## Results

50 patients attended the clinic, 41 (82%) patients had RA (39 F:2 M, mean age 60.1±11.2 years & mean disease duration 19.2±10.7 years). Non-RA patients excluded from subsequent analysis. At FU 22 (53.7%) patients had surgery, 14 (34.1%) didn’t, 4 (9.8%) were listed & 1 died prior to surgery. 26 operations were performed on 22 patients; 23 (88.46%) forefoot procedures, 1 rearfoot & 1 ankle. In the non surgery group; 2 were referred to another speciality, 10 declined surgery & surgery was deemed inappropriate in 2. 31 (75.6%) patients returned the questionnaire, 17 (54.8%) had surgery. In the surgery group the baseline LFIS_IF_ score was 14.4±3.0 & FU was 11.8±5.6 (t=1.8; p=0.085). Baseline LFIS_AP_ was 20.1±6.8 & FU was 16.8±8.7 (t=2.1; p=0.051). In the foot that had surgery the baseline pVAS was 58.3±23.1 & post surgery pVAS was 26.2±21.4 (t=3.8; p=0.002). In the non-surgery group the baseline LFIS_IF_ score was 12.5±4.7 & FU 13.6±5.7 (NS), LFIS_AP_ score was 21.9±8.6 & FU was 20.7±9.6 (NS). The baseline pVAS for the left foot was 33.4±24.3 & FU 46.8±30.4 (NS). The pVAS for the right foot was 56.0±27.9 & FU 51.5±32.2 (NS).

## Conclusions

In a selected group of patients surgery resulted in a significant reduction in pain with a strong trend towards improvement in foot related quality of life. There was no change in the non surgery group indicating more treatment options are required. Careful selection of patients demonstrated the benefits of combined care suggesting more specific guidelines are required to aid appropriate surgical referrals.

